# Network-based survival-associated module biomarker and its crosstalk with cell death genes in ovarian cancer

**DOI:** 10.1038/srep11566

**Published:** 2015-06-23

**Authors:** Nana Jin, Hao Wu, Zhengqiang Miao, Yan Huang, Yongfei Hu, Xiaoman Bi, Deng Wu, Kun Qian, Liqiang Wang, Changliang Wang, Hongwei Wang, Kongning Li, Xia Li, Dong Wang

**Affiliations:** 1 College of Bioinformatics Science and Technology, Harbin Medical University, Harbin, China

## Abstract

Ovarian cancer remains a dismal disease with diagnosing in the late, metastatic stages, therefore, there is a growing realization of the critical need to develop effective biomarkers for understanding underlying mechanisms. Although existing evidences demonstrate the important role of the single genetic abnormality in pathogenesis, the perturbations of interactors in the complex network are often ignored. Moreover, ovarian cancer diagnosis and treatment still exist a large gap that need to be bridged. In this work, we adopted a network-based survival-associated approach to capture a 12-gene network module based on differential co-expression PPI network in the advanced-stage, high-grade ovarian serous cystadenocarcinoma. Then, regulatory genes (protein-coding genes and non-coding genes) direct interacting with the module were found to be significantly overlapped with cell death genes. More importantly, these overlapping genes tightly clustered together pointing to the module, deciphering the crosstalk between network-based survival-associated module and cell death in ovarian cancer.

Ovarian cancer, the most lethal gynecological malignancy, is the fifth most common causes of cancer death in women^1^. Generally, ovarian cancer patients have a poor prognosis with 5-year survival rate of 43.7%, and even 63% who are diagnosed with metastasis disease only have the 5-year survival rate of 26.9%^2^ attributed to late detection and chemo-resistance. Therefore, it is of critical importance to identify clinical biomarkers responsible for monitoring ovarian cancer treatment, which may lead to development of novel therapeutic targets and eventually decrease the risk of death in ovarian cancer patients.

In recent decades, unprecedented multi-level omics data provide a convenient way to identify biomarkers for ovarian cancer with rapid development of high-throughput technologies, however, the findings in these studies still lack some successful applications for ovarian cancers so far^3^,^4^. Mostly, only rare genes without functional relationships are selected and treated as a potential biomarker of ovarian cancer^3^,^5^,^6^. Furthermore, owing to the highly clinically and genetically heterogeneous nature of cancer, several irrelevant abnormalities that are either low frequency of occurrences in all patients or rarely sufficient to cause cancer^3^,^5^,^6^,^7^ are probably not the ideal candidate considering the high reproducibility and sensitivity of biomarker. These difficulties will lead to a fatal problem influencing their application. However, considering the complex nature of the interaction between genes, single genetic abnormality can spread along the links of the complex intracellular network to alter a series of common gene products’ activities in either a direct or an indirect manner^8^,^9^,^10^. Just as Taylor IW *et al.* emphasized that changes in the biochemical wiring of oncogenic cells drives phenotypic transformations that can directly affect disease outcome^11^. Hence, it has recent emerged several network-based studies utilizing interaction information between genes in ovarian cancer^12^,^13^. However, these studies primarily fall into the category of static network analysis ignoring dynamics of network on both temporal and spatial specificity. Therefore, network-based dynamic modularity analysis for survival-associated biomarker discovery in longer-versus shorter-survival patients will provide more robust insights into preclinical therapeutic modality development on ovarian cancer.

More importantly, to a better understanding of outcome and optimal treatment of ovarian cancer, researchers are not fully satisfied with the challenges of obtaining biomarkers, thus, substantial interests have arisen to decipher their biological function. Currently, it gradually became clear that biomarker genes are mainly involved in these processes of immune, inflammatory, cell cycle and cell death^14^,^15^,^16^. Specifically for cell death, as a fundamental biological process, it plays an important role during the development, maintenance of tissue homeostasis, and elimination of damaged cells^17^,^18^. Growing evidences have shown that excessive or defective cell death contributes to a broad spectrum of human diseases, including ovarian cancer^19^,^20^,^21^. Insights into the molecular mechanisms involved in cell death will likely have important implications and offer the opportunity to target this process for therapeutic purpose^22^,^23^,^24^,^25^. However, the rational treatment design and selection are often precluded due to the lack of the elaborate wiring diagram of biomarkers and cell death. Therefore, it is necessary to dissect and decipher the crosstalk between biomarkers and cell death. Shaping this roadmap will definitely provide more benefit for a more accurate outcome prediction and personalized management of ovarian cancer.

In this paper, based on constructed weighted survival and differential co-expression network between longer- versus shorter- survival patients using survival information, protein-protein interactions (PPI) in STRING^26^ and gene expression data from TCGA ovarian cancer, we adopted a network-based approach to capture a 12-gene network module. Survival analysis showed that this module was significantly related to overall survival of patients in ovarian cancer, whose prognostic ability was further confirmed in internal and external independent datasets. To elucidate the underlying mechanisms of this module in ovarian cancer, we further explored the genes significantly regulating the 12-gene module. Functional annotation of these genes showed a close correlation with cell death. Specifically, these significantly regulatory genes direct interacting with the module were significantly overlapped with cell death genes from our miRDeathDB^27^,^28^, and HADB^29^ and DeathBase^30^. More importantly, these overlapping genes were found to be tightly clustered together pointing to the 12-gene module. These findings highlighted that deciphering the crosstalk between network-based survival-associated module and cell death in ovarian cancer not only sheds light on its mechanism of action, but may also contribute to biomarker-guided preclinical and clinical therapeutic modality development.

## Results

### Identification of network-based survival-associated module in ovarian cancer

**Figure 1:**
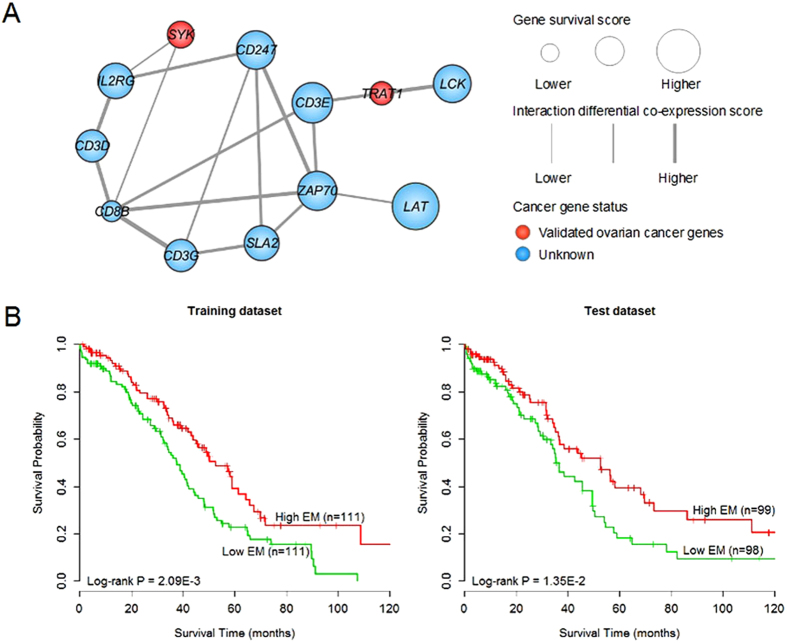
The 12-gene module biomarker and Kaplan-Meier estimates of overall survival in ovarian cancer patients according to this module biomarker. (**A**) The interaction wiring of the 12-gene module. The nodes in red or blue indicated whether the genes have been verified as being related to ovarian cancer or not, respectively. The node sizes indicated the significance of association between the genes with cancer survival. The width of edges indicated the extent of differential co-expression of two genes. (**B**) Kaplan-Meier estimates of overall survival in the training and internal test datasets.

**Figure 2:**
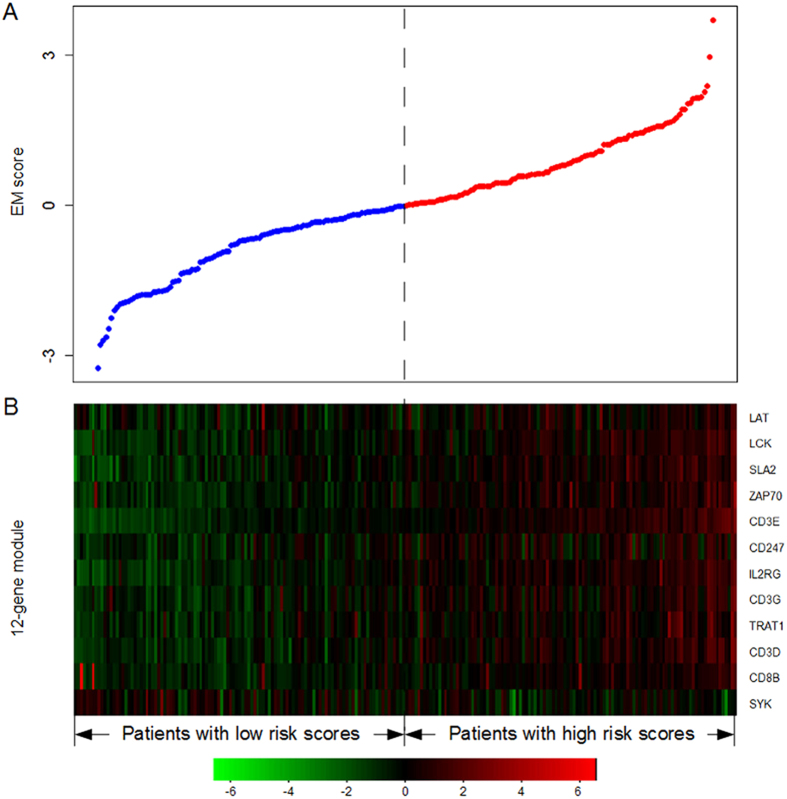
12-gene module risk score analysis of ovarian cancer. (**A**) The distribution of the 12-gene module risk score. Patients were divided into a high-risk group (Red) or a low-risk group (Blue) using the median risk score as the cutoff point. (**B**) Heatmap of the module genes’ expression profiles. Rows and columns represented genes and patients, respectively.

To identify module biomarkers of ovarian cancer, we first adopted a network-based simulated annealing approach to search putative modules by integrating survival information, PPI network and gene expression. Under the criteria with module score ranked in the top 1% (module score > 6.15) and p < 0.01, a total of 71 modules were identified in the constructed survival and differential co-expression PPI network between longer- versus shorter- survival patients. Then, for each module, we evaluated its predictive ability for survival of ovarian cancer patients, as described in the Materials and Methods. Notably, 27 of 71 modules were found to be significantly associated with overall survival of ovarian cancer patients in the training dataset (p < 0.1). Among all the survival-associated modules, the predictive ability of only a 12-gene module (Fig. 1, Table 1 and Supplementary Table S1), was further confirmed in independent internal dataset (In training dataset, log-rank p = 2.09E-3; In test dataset, log-rank p = 0.014). Gene Ontology functional annotation on the 12-gene module was presented in Supplementary Table S2. The distribution of the module genes’ risk scores and heatmap of the module genes’ expression profiles were shown in Fig. 2.

By using Cytoscape^31^, interaction wiring of the module genes was visualized in Fig. 1A. And it can be clearly seen that the module genes exhibited a context-specific PPI pattern, reflecting the dynamic feature of module facing to different malignant extent of ovarian cancer patients. Following this, the clinical relevance of the module genes was further examined. As shown in Table 1, except for CD8B, TRAT1 and SYK, all the other module genes were found to be significantly associated with survival of ovarian cancer patients (p < 0.05). More importantly, the CD8B, TRAT1 and SYK genes were here included in this module because of owning the larger differential co-expression with their interaction neighbors, the survival-associated genes between longer- and shorter- survival patients (Supplementary Table S1). For instance, CD8B through interacting with the survival-associated CD3G, CD3E, CD3D and ZAP70 genes was recruited in this module. Although no significant survival association was observed in these three genes, their potential biological or clinical relevance were confirmed by the evidences excavated from related researches. Especially, cancer genes, TRAT1 and SYK, have been validated as being significantly associated with lethal ovarian cancer, driving malignant transformation of ovarian cancer^32^,^33^,^34^. Taken together, the network-based module analysis has the ability to provide a deeper understanding of the characteristics of biomarker beyond biomarker discovery.

### Independent external validation of the 12-gene module biomarker in ovarian cancer

**Figure 3:**
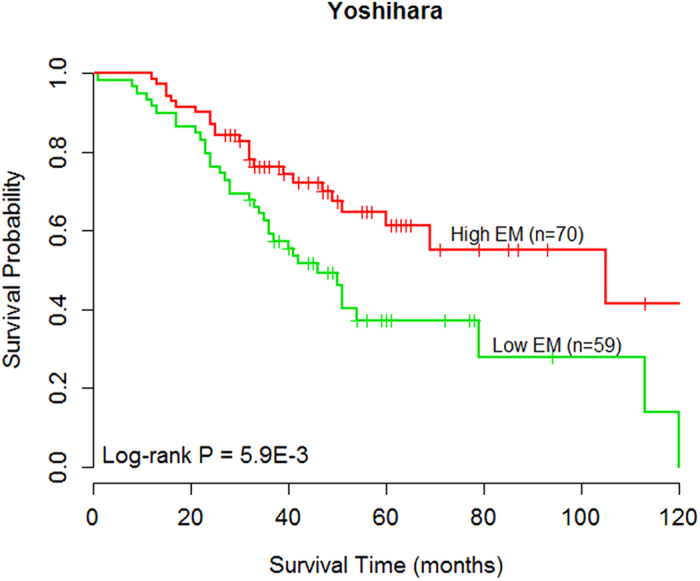
Kaplan-Meier estimates of overall survival in the independent external Yoshihara dataset according to the 12-gene module biomarker.

To further assess the predictive ability of the 12-gene module biomarker, we here used an independent external data for ovarian cancer^35^, in which only those patients with advanced-stage, high-grade ovarian serous cancer were analyzed. After dividing the patients into two subgroups according to the median value of EM scores in training dataset, we found that the 12-gene module was significantly related to overall survival of ovarian cancer patients using the log-rank test (log-rank p = 5.9E-3; Fig. 3). The hazard ratio of high-risk versus low-risk groups was 0.49 (95% CI: 0.29–0.82, p = 7.12E-3). Specifically, the patients with low EM scores resided in the high-risk group with a shorter survival. The median survival time for low-risk group was 105 months, whereas the median survival time for high-risk group was only 46 months. To further verify the correlation between the 12-gene module and survival, the predictive ability of the module in the independent external data was further confirmed and presented in Supplementary Table S3.

### Prognostic value of the 12-gene module for assessing clinical outcome of ovarian cancer

After further adjusting for age, grade, stage, and residual tumor size, as shown in Table 2, the univariate and multivariate analysis indicated that the 12-gene module biomarker, as an independent risk factor, was significantly associated with overall survival of ovarian cancer patients in the training (HR = 0.47, 95% CI: 0.38–0.73, p = 1.58E-4) and internal test (HR = 0.63, 95% CI: 0.41-0.97, p = 0.038) data. In addition, multivariate analysis also demonstrated that the designation of high- and low-risk groups remained statistically significant in the independent external data (HR = 0.51, 95% CI: 0.30-0.88, p = 0.014). Taken together, these analyses demonstrated the capacity of the 12-gene module biomarker to add value in a prognostic setting.

### Crosstalk between the 12-gene module and cell death

Considering that the 12-gene module biomarker only provided a starting point for improving the decision making process, further research will be necessary to elucidate the underlying mechanisms of this module in ovarian cancer. Here, we examined the network wiring around the module genes and extracted their significant interacting neighbors. In total, 151 significantly regulating genes including miRNA genes were identified (p < 0.1, hypergeometric test; Supplementary Table S4) as described in Materials and Methods. Then, annotation enrichment analysis using DAVID^36^,^37^ showed that 146 of 151 genes were annotated to 51 KEGG pathways (all p < 0.1), and detailed description of these pathways was presented in Supplementary Table S5. Specifically, the above enrichment analysis demonstrated a close correlation between the regulating genes and cell death for apoptosis pathway (p = 4.2E-6).

**Figure 4:**
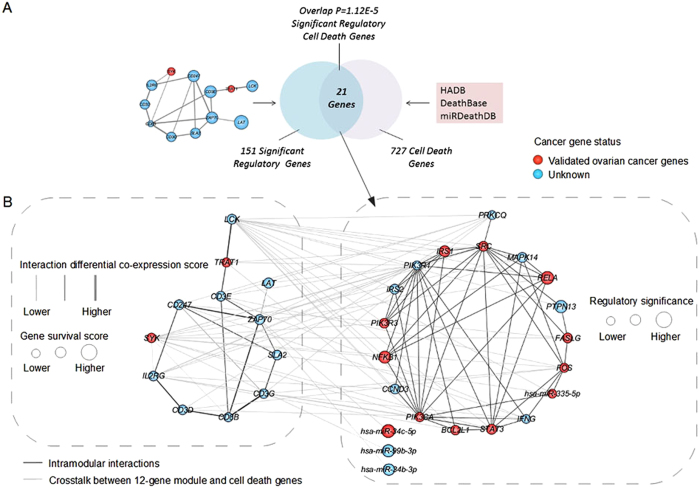
Overlap and wiring diagram between the regulatory genes and cell death genes. (**A**) Venn plot, showing a nonrandom amount of overlap between the 151 significantly regulatory genes and 727 cell death genes. (**B**) The interaction wiring of 21 cell death genes and their wiring connections on the 12-gene module. The nodes in red or blue indicated whether the genes have been verified as being related to ovarian cancer or not, respectively. The node sizes indicated the significance of the genes in regulating the 12-gene module.

Notably, cell death, an established cancer hallmark, might serve as a promising candidate in prevention and treatment of ovarian cancer. We further explored the relationships between the 12-gene module biomarker and cell death genes from our miRDeathDB^27^,^28^, and HADB^29^ and DeathBase^30^. A nonrandom amount of overlap was observed between the 151 significantly regulating genes and 727 cell death genes (p = 1.12E-5, hypergeometric test; Fig. 4A), suggesting potential clinical benefit for tumor suppression via regulating cell death. For example, STAT3, contributing to oncogenesis by inhibition of apoptosis, interacts with LCK leading to T-cell transformation by Herpesvirus saimiri (HVS)^38^. Specifically, 18 of 21 overlapping genes were found to be tightly clustered together pointing to the module biomarker (Fig. 4B). Moreover, majority of the overlapping genes were known to be cancer genes, whose close association with ovarian cancer have been confirmed as presented in detail in Table 3. For example, BCL2L1, as a key protein in regulating programmed cell death or apoptosis, was found to be dysregulated in ovarian cancer cell lines and specimens that promoted cancer progression^39^. The hsa-miR-335-5p was regarded as an invasion suppressor, whose dysregulation drove cancer transformation by targeting Bcl-w^24^,^40^.

Taken together, these results provided the additional evidence to support our findings from the aspect of biological importance, demonstrating that based on integrating survival information and differential co-expression between longer- and shorter- survival patients, the network-based survival-associated module biomarker has guidance for the treatment of ovarian cancer, excepting for the diagnosis of ovarian cancer.

## Discussion

Ovarian cancer, as a complex disease, is characterized by dysregulation of multiple cellular functions that interact in a complex network environment^10^. Furthermore, gene intersections and their dynamic wiring, as essential components of network, underlie the orchestration of biological processes^10^, hence, it is reasonable to perform network-based dynamic modularity analysis for biomarker discovery. Different from the traditional network-based analyses that usually ignore the patients’ survival hazards or the correlations existing between gene expressions^41^,^42^, we here facilitated the dynamic responded-intersections based on survival and differential co-expression PPI network between longer- and shorter-survival patients and identified a 12-gene module biomarker for ovarian cancer, and further confirmed its predictive ability in internal and external independent datasets. Despite the 12-gene module biomarker was shown to be an independent risk factor for ovarian cancer from age, grade, stage, and residual tumor size, the influence of drug on the survival of ovarian cancer patients should be further excluded. Nevertheless, this analysis is consistent with a clinical viewpoint that the rationale behind the biomarker discovery is to find robust and effective biomarker, given that modules play a central role in maintaining network stability.

When the transition from normal state into disease state, network will subject to many forms of disruption and network modules as response element of the disease, which can confer cellular functions. Thus, the functional analysis of module biomarker will enhance our understanding of the underlying mechanisms of the disease. As for the identified 12-gene module biomarker, we further deciphered the underlying biological mechanisms of the biomarker and dissected the wiring diagram between the biomarker and cell death, and found the module genes having close interaction with cell death.

Notably, the wiring analysis of module biomarker demonstrated that its 151 regulating interacting neighbors were significantly overlapped with cell death genes, of which 21 overlapping cell death genes interacted closely with the 12-gene module. More specifically, 4 of 21 overlapped cell death genes were miRNAs, which was overwhelming (4/5 = 80%) in the significant regulatory miRNAs, implying that cell death related non-coding RNAs might play an important role in regulating the 12-gene module. And 18 overlapped cell death genes clustered together pointing to the 12-gene module, implying the cooperative behavior for the benefit to increase evades and prevents cell death. These analyses begin to bridge the gap between cancer diagnosis and treatment and pave a clear path from cancer diagnosis to treatment.

In conclusion, our analyses demonstrated the effectiveness and robustness of network-based module analysis for biomarker discovery by integrating survival information and differential co-expression between longer- and shorter- survival patients, highlighting the importance of functional analysis in understanding biomarker prediction and monitoring treatment. Especially, the wiring diagram discovery between biomarker and cell death has made an important step towards transforming from preclinical to clinical assessments.

## Methods

### Gene expression and clinical data

Gene expression data (Level 3) generated by Agilent platform and clinical data of 419 patients with advanced-stage (stages III and IV), high-grade (grades 3 and 4) ovarian serous cystadenocarcinoma were downloaded from TCGA repository (http://cancergenome.nih.gov/). Notably, this dataset was divided into two subsets: one training set consisting of Batches 9, 11–15 and 17 and one test set consisting of Batches 18–19, 21–22, 24, 27 and 40, as described in detail in Table 4. Separately for each set, the patients were further stratified into the longer- and shorter- survival groups according to the criteria that a 37-month median survival time in ovarian cancer identified by Macmillan Cancer Support (http://www.macmillan.org.uk/) derived from the researches of the Cancer Research UK Cancer Survival Group at the London School of Hygiene and Tropical Medicine. Additionally, a cohort of 129 ovarian cancer patients from GEO database (Accession No. GSE32062)^35^ was used as an independent external test set. Microarray data was median-normalized and replicate genes were combined by averaging their expression values^43^,^44^.

### Protein-protein interactions (PPI), RNA-protein interactions and cell death genes

Protein-protein interactions were retrieved from STRING v9.1^26^. To minimize the impact of network size, only high-confidence interactions with String-score ≥0.90 were extracted to construct network, involving 76, 709 interactions^12^. RNA-protein interactions were integrated from our RAID^45^ and miRTarBase^46^ databases.

Cell death genes were manually reviewed from literatures and collected from our miRDeathDB^27^,^28^, and HADB^29^ and DeathBase^30^ databases. After removing the redundant and unrecognized genes, a total of 727 cell death genes were used for subsequent analysis.

### Construction of weighted PPI network

A weighted PPI network was constructed, in which each node (gene 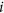) was assigned a weight, 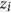, on the basis of its association with survival of patients using Cox proportional hazards regression model, as follows:


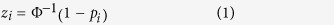


where 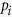 represented the significance of association between each gene expression and survival of patients, calculated from univariable Cox proportional hazards regression model. 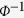 represented the inverse standard normal cumulative distribution function (CDF)^41^,^47^. Thus, 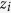 followed a standard normal distribution, with a smaller *p*-value corresponding to a larger *z*-score value.

And each edge (interaction 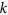) was assigned a weight, 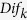, on the basis of its degree of differential co-expression of a pair genes (genes 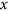 and 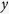) between longer- and shorter- survival samples, as follows:

First, Pearson correlation coefficient 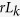 (or 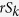) of genes 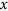 and 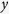 between patients in longer-survival group (or shorter-survival group) in the training dataset was calculated as


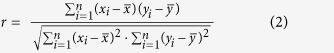


where 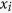 and 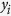 represented the expression levels of gene 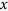 and gene 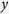 in patient 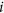 of longer-survival group (or shorter-survival group); 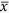 and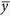 represented the average expression levels of gene 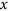 and gene 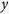 in longer-survival group (or shorter-survival group); 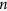 represented the number of patients in longer-survival group (or shorter-survival group).

Second, the Pearson correlation coefficient (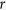) was further transformed into *z*-score value by using Fisher’s Z transformation


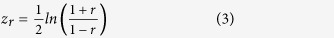


Then, the degree of differential co-expression of this pair genes (genes 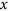 and 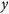) between longer- and shorter-survival groups, 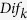, was calculated as


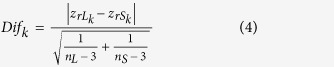


where 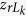 and 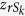 represented the transformed Pearson correlation coefficients in longer- and shorter-survival groups, respectively; 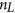 and 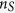 represented the numbers of patients in longer- and shorter-survival groups, respectively.

### Identification of network-based modules

As we known, the problem of finding the maximal-scoring connected module was NP-hard. To solve this problem and obtain the globally optimal solution, a simulated annealing algorithm^47^,^48^ was here introduced to search candidate modules in the weighted network. For each iteration 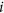, the highest-scoring module, denoted as 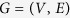, was scored by the following formula^49^:


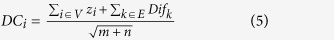


where 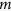 represented the number of genes (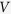) and 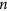 represented the number of interactions (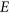) in module 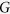.

Those modules overlapping to a very high extent, more than 80%, in comparison to their sizes were further merged concurrently rather than sequentially in order to avoid recalculating the overlap of modules^50^,^51^.

To exclude the effect of module sizes on their scores, we randomly sampled gene sets of size 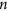, a permutation experiment using random resampling of 10,000 times was performed to estimate the score mean 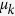 and standard deviation 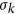, and then 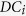 was further adjusted as follows:


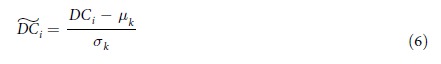


### Survival analysis and module biomarker selection

Survival curves were estimated by the Kaplan-Meier method and compared with log-rank test. Univariate and multivariate survival analyses were performed using the Cox proportional hazard model.

For each candidate module, we calculated an eigengene of the module (EM) value for each sample as the weighted average of gene expression levels of their first principal components^52^ and then assessed its predictive ability as a predictor of survival after dividing the patients into two subgroups based on the median value of EM values.

### Regulating genes identification

According to PPI or RNA-protein interactions, genes direct interacting with module genes were examined by using hypergeometric test, and those genes with p < 0.1 were defined as significant regulating genes.

## Additional Information

**How to cite this article**: Jin, N. *et al.* Network-based survival-associated module biomarker and its crosstalk with cell death genes in ovarian cancer. *Sci. Rep.* **5**, 11566; doi: 10.1038/srep11566 (2015).

## Supplementary Material

Supplementary Table S1

Supplementary Table S2

Supplementary Table S3

Supplementary Table S4

Supplementary Table S5

## Figures and Tables

**Table 1:** Summary of the 12-genemodule identified in ovarian cancer.

Gene symbol	Entrez ID	Gene name	Reference	COX p value
CD247	919	CD247 molecule	-	1.43E-3
CD3D	915	CD3d molecule, delta (CD3-TCR complex)	-	0.012
CD3E	916	CD3e molecule, epsilon (CD3-TCR complex)	-	6.86E-4
CD3G	917	CD3g molecule, gamma (CD3-TCR complex)	-	0.012
CD8B	926	CD8b molecule	-	0.17
IL2RG	3561	Interleukin 2 receptor, gamma	-	4.29E-3
LAT	27040	Linker for activation of T cells	-	4.47E-5
LCK	3932	LCK proto-oncogene, Src family tyrosine kinase	-	1.20E-3
SLA2	84174	Src-like-adaptor 2	-	5.20E-3
SYK	6850	Spleen tyrosine kinase	34	0.053
TRAT1	50852	T cell receptor associated Transmembrane adaptor 1	33	0.11
ZAP70	7535	Zeta-chain (TCR) associated protein kinase 70kDa	-	1.16E-3

**Table 2:** Univariate and multivariate Cox regression analysis of the 12-gene module biomarker in ovarian cancer datasets.

Variables	Univariate model		Multivariate model	
	HR(95% CI)	P value	HR(95% CI)	P value
Training dataset (n = 222)				
12-gene module				
Low	1 (reference)		1 (reference)	
High	0.57 (0.40, 0.82)	2.38E-3	0.47 (0.32, 0.70)	1.58E-4
Age	1.50 (1.05, 2.14)	0.03	1.80 (1.23, 2.65)	2.69E-3
Stage				
III	1 (reference)		1 (reference)	
IV	1.25 (0.77, 2.01)	0.37	1.00 (0.61, 1.64)	0.99
Grade				
G3	1 (reference)		1 (reference)	
G4	1.35 (0.19, 9.70)	0.77	1.21 (0.16, 8.83)	0.85
Residual tumor size				
0-10 mm	1 (reference)		1 (reference)	
>10 mm	1.34 (0.89, 2.02)	0.16	1.32 (0.87, 2.00)	0.19
Test dataset (n = 197)				
12-gene module				
Low	1 (reference)		1 (reference)	
High	0.60 (0.40, 0.90)	0.02	0.63 (0.41, 0.97)	0.04
Age	1.22 (0.81, 1.85)	0.34	1.27 (0.81, 1.97)	0.30
Stage				
III	1 (reference)		1 (reference)	
IV	1.28 (0.75, 2.20)	0.37	1.35 (0.71, 2.54)	0.36
Residual tumor size				
0-10 mm	1 (reference)		1 (reference)	
>10 mm	1.50 (0.77, 2.93)	0.24	1.64 (0.82, 3.29)	0.16
Yoshihara (n = 129)				
12-gene module				
Low	1 (reference)		1 (reference)	
High	0.49 (0.29, 0.82)	7.12E-3	0.51 (0.30, 0.88)	0.01
Stage				
III	1 (reference)		1 (reference)	
IV	1.47 (0.86, 2.52)	0.16	1.24 (0.71, 2.17)	0.45

Note: Two-sided p values were derived from the Cox proportional hazards model using all variables in the table. HR indicated hazard ratio. CI denoted confidence interval.

**Table 3:** A detailed description of the 21 significantly regulatory cell death genes.

Gene symbol	Entrez gene/miRBase ID	Gene name	Reference	P value
BCL2L1	598	BCL2-like 1	39	0.015
CCND3	896	Cyclin D3	-	0.022
FASLG	356	Fas ligand (TNF superfamily, member 6)	53	0.023
FOS	2353	FBJ murine osteosarcoma viral Oncogene homolog	54	5.19E-3
IFNG	3458	Interferon, gamma	-	6.34E-4
IRS1	3667	Insulin receptor substrate 1	55	0.047
IRS2	8660	Insulin receptor substrate 2	-	0.022
MAPK14	1432	Mitogen-activated protein kinase 14	-	0.026
NFKB1	4790	Nuclear factor of kappa light polypeptide gene enhancer in B-cells1	56	0.076
PIK3CA	5290	Phosphatidylinositol-4,5-bisphosphate 3-kinase, catalytic subunit alpha	57	9.87E-3
PIK3R1	5295	Phosphoinositide-3-kinase, regulatory subunit 1 (alpha)	-	1.34E-5
PIK3R3	8503	Phosphoinositide-3-kinase, regulatory subunit 3 (gamma)	58	0.020
PRKCQ	5588	Protein kinase C, theta	-	3.33E-9
PTPN13	5783	Protein tyrosine phosphatase, non-receptor type 13 (APO-1/CD95 (Fas)-associated phosphatase)	-	0.082
RELA	5970	V-rel avian reticuloendotheliosis viral oncogene homolog A	59	0.091
SRC	6714	SRC proto-oncogene, non-receptor tyrosine kinase	60	0.023
STAT3	6774	Signal transducer and activator of transcription 3 (acute-phase response factor)	61,62	0.028
	hsa-miR-335-5p		24,40	6.52E-4
	hsa-miR-34b-3p		-	0.063
	hsa-miR-34c-5p		63	0.097
	hsa-miR-99b-3p		-	0.078

**Table 4:** Clinical characteristics of patients with advanced-stage, high-grade TCGA ovarian serous cystadenocarcinoma.

Characteristic	Training dataset (n = 222)		Characteristic	Test dataset (n = 197)	
	No. of patients	Median (month)		No. of patients	Median (month)
Age (median 59.27, range 34.99 - 87.47)			Age (median 59.75, range 30.54 - 87.61)		
<59.27	110	50.3	<59.75	95	49.5
≥59.27	111	34.4	≥59.75	95	36.3
Stage			Stage		
III	186	44.0	III	171	42.6
IV	36	42.1	IV	26	31.7
Grade			Grade		
3	221	42.1	3	197	42.6
4	1	44.7	4	/	/
Residual tumor size			Residual tumor size		
0–10 mm	148	41.6	0–10 mm	167	42.6
>10 mm	52	33.9	>10 mm	18	34.6

Note: Patients with no macroscopic disease were classified into the 0–10-mm group. Median denoted mediansurvival time.
