# Supplementary material for: Network-based survival-associated module biomarker and its crosstalk with cell death genes in ovarian cancer
Source: Sci Rep. 2015 Jun 23;5:11566. doi: 10.1038/srep11566 (PMC4477367; doi:10.1038/srep11566)
Supplement: Supplementary Table S1 [file srep11566-s1.pdf]

Network-based survival-associated module biomarker and its crosstalk with cell death genes in ovarian cancer

Nana Jin*, Hao Wu*, Zhengqiang Miao*, Yan Huang*, Yongfei Hu, Xiaoman Bi, Deng Wu, Kun Qian, Liqiang Wang, Changliang Wang, Hongwei Wang, Kongning Li, Xia Li, Dong Wang

Authors' affiliations: College of Bioinformatics Science and Technology, Harbin Medical University, Harbin, China

Corresponding authors: Dong Wang, College of Bioinformatics Science and Technology, Harbin Medical University, Harbin 150086, China. Phone: +86 045186615933; Fax: +86 045186615933; E-mail: wangdong@ems.hrbmu.edu.cn; and Xia Li, E-mail: lixia@hrbmu.edu.cn; and Kongning Li, E-mail: kongningli@hotmail.com; and Hongwei Wang, E-mail: bioccwhw@126.com

* These authors contributed equally to this work.

Supplementary Table S1: Interaction differential co-expression scores in the 12-gene module

Interactor1	Interactor2	Interaction differential co-expression score
CD8B	CD3G	2.32
TRAT1	LCK	1.84
ZAP70	CD247	1.79
CD8B	ZAP70	1.76
CD8B	CD3D	1.68
IL2RG	CD3D	1.64
CD3G	SLA2	1.29
CD247	IL2RG	1.24
CD3E	TRAT1	1.22
CD3E	ZAP70	1.21
CD8B	CD3E	1.09
CD247	SLA2	1.09
SLA2	ZAP70	1.02
ZAP70	LAT	0.46
CD3G	CD247	0.44
SYK	CD8B	0.14
SYK	IL2RG	0.06
