# Supplementary material for: Network-based survival-associated module biomarker and its crosstalk with cell death genes in ovarian cancer
Source: Sci Rep. 2015 Jun 23;5:11566. doi: 10.1038/srep11566 (PMC4477367; doi:10.1038/srep11566)
Supplement: Supplementary Table S2 [file srep11566-s2.pdf]

# Network-based survival-associated module biomarker and its crosstalk with cell death genes in ovarian cancer

Nana Jin*, Hao Wu*, Zhengqiang Miao*, Yan Huang*, Yongfei Hu, Xiaoman Bi, Deng Wu, Kun Qian, Liqiang Wang, Changliang Wang, Hongwei Wang, Kongning Li, Xia Li, Dong Wang

Authors' affiliations: College of Bioinformatics Science and Technology, Harbin Medical University, Harbin, China

Corresponding authors: Dong Wang, College of Bioinformatics Science and Technology, Harbin Medical University, Harbin 150086, China. Phone: +86 045186615933; Fax: +86 045186615933; E-mail: wangdong@ems.hrbmu.edu.cn; and Xia Li, E-mail: lixia@hrbmu.edu.cn; and Kongning Li, E-mail: kongningli@hotmail.com; and Hongwei Wang, E-mail: bioccwhw@126.com

* These authors contributed equally to this work.

**Supplementary Table S2:** Gene Ontology functional annotation on the 12-gene module using DAVID

| ID | Gene Name | GOTERM_BP |
|---|---|---|
| CD247 | CD247 molecule | GO:0002376~immune system process,GO:0051704~multi-organism process,GO:0065007~biological regulation |
| CD3D | CD3d molecule, delta (CD3-TCR complex) | GO:0002376~immune system process,GO:0009987~cellular process,GO:0032501~multicellular organismal process,GO:0032502~developmental process |
| CD3E | CD3e molecule, epsilon (CD3-TCR complex) | GO:0002376~immune system process,GO:0009987~cellular process,GO:0016043~cellular component organization,GO:0032501~multicellular organismal process,GO:0032502~developmental process,GO:0044085~cellular component biogenesis,GO:0065007~biological regulation |
| CD3G | CD3g molecule, gamma (CD3-TCR complex) | GO:0002376~immune system process,GO:0009987~cellular process,GO:0016043~cellular component organization,GO:0032502~developmental process,GO:0044085~cellular component biogenesis,GO:0051179~localization,GO:0051234~establishment of localization,GO:0065007~biological regulation |
| CD8B | CD8b molecule | GO:0002376~immune system process,GO:0009987~cellular process,GO:0050896~response to stimulus,GO:0065007~biological regulation |
| SLA2 | Src-like-adaptor 2 | GO:0002376~immune system process,GO:0009987~cellular process,GO:0050896~response to stimulus,GO:0065007~biological regulation |
| TRAT1 | T cell receptor associated transmembrane adaptor 1 | GO:0002376~immune system process,GO:0050896~response to stimulus,GO:0065007~biological regulation |
| LCK | lymphocyte-specific protein tyrosine kinase | GO:0002376~immune system process,GO:0008152~metabolic process,GO:0009987~cellular process,GO:0016265~death,GO:0032501~multicellular organismal process,GO:0032502~developmental process,GO:0050896~response to stimulus,GO:0051179~localization,GO:0051234~establishment of localization,GO:0051704~multi-organism process,GO:0065007~biological regulation |
| SYK | spleen tyrosine kinase | GO:0002376~immune system process,GO:0008152~metabolic process,GO:0009987~cellular process,GO:0016043~cellular component organization,GO:0022610~biological adhesion,GO:0032501~multicellular organismal process,GO:0032502~developmental process,GO:0040011~locomotion,GO:0044085~cellular component biogenesis,GO:0050896~response to stimulus,GO:0051179~localization,GO:0051234~establishment of localization,GO:0051704~multi-organism process,GO:0065007~biological regulation |

| | | |
|---|---|---|
| ZAP70 | zeta-chain (TCR) associated protein kinase 70kDa | GO:0002376~immune system process,GO:0008152~metabolic process,GO:0009987~cellular process,GO:0032501~multicellular organismal process,GO:0032502~developmental process,GO:0050896~response to stimulus,GO:0065007~biological regulation |
