# Supplementary material for: Network-based survival-associated module biomarker and its crosstalk with cell death genes in ovarian cancer
Source: Sci Rep. 2015 Jun 23;5:11566. doi: 10.1038/srep11566 (PMC4477367; doi:10.1038/srep11566)
Supplement: Supplementary Table S3 [file srep11566-s3.pdf]

Network-based survival-associated module biomarker and its crosstalk with cell death genes in ovarian cancer

Nana Jin[*], Hao Wu[*], Zhengqiang Miao[*], Yan Huang[*], Yongfei Hu, Xiaoman Bi, Deng Wu, Kun Qian, Liqiang Wang, Changliang Wang, Hongwei Wang, Kongning Li, Xia Li, Dong Wang

Authors' affiliations: College of Bioinformatics Science and Technology, Harbin Medical University, Harbin, China

Corresponding authors: Dong Wang, College of Bioinformatics Science and Technology, Harbin Medical University, Harbin 150086, China. Phone: +86 045186615933; Fax: +86 045186615933; E-mail: wangdong@ems.hrbmu.edu.cn; and Xia Li, E-mail: lixia@hrbmu.edu.cn; and Kongning Li, E-mail: kongningli@hotmail.com; and Hongwei Wang, E-mail: bioccwhw@126.com

[*] These authors contributed equally to this work.

Supplementary Table S3: Cox score of the 12-gene module in Yoshihara

Gene symbol	Entrez ID	COX p value in Yoshihara
CD247	919	1.16E-3
CD3D	915	3.83E-4
CD3E	916	0.069
CD3G	917	2.4E-3
CD8B	926	0.026
IL2RG	3561	4.71E-4
LAT	27040	0.030
LCK	3932	9.57E-4
SLA2	84174	1.46E-3
SYK	6850	0.21
TRAT1	50852	9.52E-4
ZAP70	7535	1.99E-3
