# Supplementary material for: Network-based survival-associated module biomarker and its crosstalk with cell death genes in ovarian cancer
Source: Sci Rep. 2015 Jun 23;5:11566. doi: 10.1038/srep11566 (PMC4477367; doi:10.1038/srep11566)
Supplement: Supplementary Table S4 [file srep11566-s4.pdf]

Network-based survival-associated module biomarker and its crosstalk with cell death genes in ovarian cancer

Nana Jin*, Hao Wu*, Zhengqiang Miao*, Yan Huang*, Yongfei Hu, Xiaoman Bi, Deng Wu, Kun Qian, Liqiang Wang, Changliang Wang, Hongwei Wang, Kongning Li, Xia Li, Dong Wang

Authors' affiliations: College of Bioinformatics Science and Technology, Harbin Medical University, Harbin, China

Corresponding authors: Dong Wang, College of Bioinformatics Science and Technology, Harbin Medical University, Harbin 150086, China. Phone: +86 045186615933; Fax: +86 045186615933; E-mail: wangdong@ems.hrbmu.edu.cn; and Xia Li, E-mail: lixia@hrbmu.edu.cn; and Kongning Li, E-mail: kongningli@hotmail.com; and Hongwei Wang, E-mail: bioccwhw@126.com

* These authors contributed equally to this work.

Supplementary Table S4: Summary of the 151 significant regulating genes pointing to the 12-gene module

Gene symbol	Entrez gene / miRBase ID	P value
ITK	3702	5.90E-13
CD4	920	5.14E-12
GRAP2	9402	6.47E-12
LCP2	3937	1.01E-11
FYB	2533	5.94E-11
MAP4K1	11184	2.83E-10
IL2RB	3560	6.63E-10
PTPN6	5777	2.60E-09
PRKCQ	5588	3.33E-09
PRF1	5551	1.42E-08
IL2RA	3559	1.65E-08
VAV1	7409	2.02E-08
B2M	567	6.05E-08
EOMES	8320	8.08E-08
SH3BP2	6452	1.89E-07
FYN	2534	2.97E-07
CD86	942	4.29E-07
IL2	3558	4.74E-07
CD80	941	5.14E-07
CSK	1445	8.68E-07
CBL	867	1.17E-06
SHC1	6464	2.92E-06
PTPRC	5788	5.36E-06
PIK3R1	5295	1.34E-05
PAG1	55824	2.43E-05
PLCG1	5335	3.87E-05
BLNK	29760	3.88E-04
PLCG2	5336	4.89E-04
IFNG	3458	6.35E-04
	hsa-miR-335-5p	6.52E-04
PDCD1	5133	6.97E-04
TYROBP	7305	8.58E-04
PTK2B	2185	9.81E-04
RGS1	5996	9.81E-04
GP6	51206	1.06E-03
JUN	3725	1.12E-03
DOK2	9046	1.72E-03
FGR	2268	1.85E-03
DBNL	28988	2.25E-03
HCK	3055	2.59E-03
JAK3	3718	2.59E-03

BTK	695	3.07E-03
VAV2	7410	3.34E-03
PTPN11	5781	3.47E-03
PIK3CB	5291	4.05E-03
DAPP1	27071	5.00E-03
MAPK11	5600	5.01E-03
FOS	2353	5.19E-03
HLA-DRB5	3127	5.41E-03
BLK	640	5.83E-03
VAV3	10451	5.83E-03
YES1	7525	6.10E-03
FCER1G	2207	6.27E-03
CBLB	868	7.67E-03
GRB2	2885	8.06E-03
CD19	930	8.16E-03
SMPD1	6609	8.66E-03
IL12RB1	3594	8.66E-03
NCR1	9437	8.91E-03
PTPRH	5794	8.91E-03
PIK3CA	5290	9.87E-03
STAM2	10254	1.03E-02
SNCA	6622	1.03E-02
PXN	5829	1.20E-02
JAK1	3716	1.22E-02
IL15RA	3601	1.33E-02
IL12B	3593	1.38E-02
BCL2L1	598	1.50E-02
LAT2	7462	1.77E-02
CARD9	64170	1.77E-02
CD244	51744	1.77E-02
FCGR3A	2214	1.77E-02
PTPN22	26191	1.77E-02
IKZF3	22806	1.77E-02
SKAP1	8631	1.77E-02
GAB2	9846	1.90E-02
PIK3R3	8503	1.97E-02
IRS2	8660	2.19E-02
CCND3	896	2.19E-02
IBTK	25998	2.20E-02
KLRG1	10219	2.20E-02
UNC119	9094	2.20E-02
SLC4A1	6521	2.20E-02
CD48	962	2.20E-02
SRC	6714	2.25E-02

FASLG	356	2.27E-02
MAPK14	1432	2.55E-02
GRAP	10750	2.62E-02
SH2D2A	9047	2.62E-02
DOK1	1796	2.62E-02
TNFRSF9	3604	2.62E-02
FCGR2A	2212	2.62E-02
CD69	969	2.62E-02
HCLS1	3059	2.62E-02
STAT3	6774	2.79E-02
BCAR1	9564	2.89E-02
TREM2	54209	3.05E-02
CD5	921	3.05E-02
TNFRSF18	8784	3.05E-02
TDGF1	6997	3.05E-02
MED28	80306	3.05E-02
SOCS3	9021	3.05E-02
TYK2	7297	3.39E-02
WIPF1	7456	3.47E-02
SH3BP5	9467	3.47E-02
IL7R	3575	3.47E-02
NCK1	4690	3.65E-02
NFKBIA	4792	3.82E-02
KIR2DL3	3804	3.88E-02
CD22	933	3.88E-02
CD72	971	3.88E-02
CD2	914	3.88E-02
ADAM15	8751	3.88E-02
PIK3AP1	118788	4.30E-02
SELPLG	6404	4.30E-02
IRS1	3667	4.73E-02
PIK3R2	5296	4.83E-02
NRAS	4893	4.92E-02
CD28	940	5.11E-02
APBB1IP	54518	5.51E-02
IL15	3600	5.91E-02
DLG1	1739	6.30E-02
SH2B3	10019	6.30E-02
PIK3R5	23533	6.30E-02
CD38	952	6.30E-02
	hsa-miR-34b-3p	6.30E-02
CSF2RB	1439	6.30E-02
FCER1A	2205	6.30E-02
SOS1	6654	6.68E-02

EPHA1	2041	6.69E-02
CTLA4	1493	7.08E-02
SPHK1	8877	7.08E-02
EPHA5	2044	7.08E-02
GZMB	3002	7.46E-02
SOCS2	8835	7.46E-02
FER	2241	7.46E-02
	hsa-miR-615-3p	7.51E-02
NFKB1	4790	7.59E-02
	hsa-miR-99b-3p	7.84E-02
CISH	1154	7.84E-02
STAT1	6772	7.90E-02
ITGA2B	3674	8.22E-02
PTPN13	5783	8.22E-02
IL7	3574	8.59E-02
CD79B	974	8.59E-02
EPHB1	2047	8.59E-02
RELA	5970	9.14E-02
EPHA7	2045	9.32E-02
	hsa-miR-34c-5p	9.69E-02
IL4R	3566	9.69E-02
EPHA3	2042	9.69E-02
