# Supplementary material for: Network-based survival-associated module biomarker and its crosstalk with cell death genes in ovarian cancer
Source: Sci Rep. 2015 Jun 23;5:11566. doi: 10.1038/srep11566 (PMC4477367; doi:10.1038/srep11566)
Supplement: Supplementary Table S5 [file srep11566-s5.pdf]

Network-based survival-associated module biomarker and its crosstalk with cell death genes in ovarian cancer

Nana Jin*, Hao Wu*, Zhengqiang Miao*, Yan Huang*, Yongfei Hu, Xiaoman Bi, Deng Wu, Kun Qian, Liqiang Wang, Changliang Wang, Hongwei Wang, Kongning Li, Xia Li, Dong Wang

Authors' affiliations: College of Bioinformatics Science and Technology, Harbin Medical University, Harbin, China

Corresponding authors: Dong Wang, College of Bioinformatics Science and Technology, Harbin Medical University, Harbin 150086, China. Phone: +86 045186615933; Fax: +86 045186615933; E-mail: wangdong@ems.hrbmu.edu.cn; and Xia Li, E-mail: lixia@hrbmu.edu.cn; and Kongning Li, E-mail: kongningli@hotmail.com; and Hongwei Wang, E-mail: bioccwhw@126.com

* These authors contributed equally to this work.

Supplementary Table S5: 51 KEGG pathways associated with significant regulating genes by gene annotation enrichment analysis using DAVID

Category	Term	Count	%	P Value	Fold Enrichment	Bonferroni	Benjamini	FDR
KEGG_PATHWAY	hsa04660:T cell receptor signaling pathway	37	25.34	7.41E-34	14.64	6.45E-32	6.45E-32	8.01E-31
KEGG_PATHWAY	hsa04630:Jak-STAT signaling pathway	35	23.97	3.93E-25	9.65	3.42E-23	1.71E-23	4.25E-22
KEGG_PATHWAY	hsa04662:B cell receptor signaling pathway	27	18.49	7.12E-25	15.38	6.20E-23	2.07E-23	7.70E-22
KEGG_PATHWAY	hsa04650:Natural killer cell mediated cytotoxicity	32	21.92	9.75E-24	10.28	8.48E-22	2.12E-22	1.05E-20
KEGG_PATHWAY	hsa04664:Fc epsilon RI signaling pathway	22	15.07	1.35E-17	12.05	1.17E-15	2.35E-16	1.46E-14
KEGG_PATHWAY	hsa04722:Neurotrophin signaling pathway	24	16.44	2.65E-15	8.27	2.32E-13	3.86E-14	2.88E-12
KEGG_PATHWAY	hsa04062:Chemokine signaling pathway	26	17.81	3.81E-13	5.94	3.31E-11	4.73E-12	4.12E-10
KEGG_PATHWAY	hsa05220:Chronic myeloid leukemia	18	12.33	4.93E-13	10.26	4.29E-11	5.36E-12	5.33E-10
KEGG_PATHWAY	hsa04012:ErbB signaling pathway	17	11.64	7.57E-11	8.35	6.58E-09	7.31E-10	8.18E-08
KEGG_PATHWAY	hsa04666:Fc gamma R-mediated phagocytosis	17	11.64	3.02E-10	7.65	2.63E-08	2.63E-09	3.27E-07
KEGG_PATHWAY	hsa04620:Toll-like receptor signaling pathway	17	11.64	7.80E-10	7.19	6.78E-08	6.17E-09	8.43E-07
KEGG_PATHWAY	hsa04670:Leukocyte transendothelial migration	18	12.33	1.02E-09	6.52	8.85E-08	7.38E-09	1.10E-06
KEGG_PATHWAY	hsa04370:VEGF signaling pathway	15	10.27	1.03E-09	8.55	8.96E-08	6.89E-09	1.11E-06
KEGG_PATHWAY	hsa05221:Acute myeloid leukemia	12	8.22	5.97E-08	8.84	5.19E-06	3.71E-07	6.45E-05
KEGG_PATHWAY	hsa05330:Allograft rejection	10	6.85	7.99E-08	11.87	6.95E-06	4.64E-07	8.64E-05
KEGG_PATHWAY	hsa05214:Glioma	12	8.22	1.46E-07	8.14	1.27E-05	7.96E-07	1.58E-04
KEGG_PATHWAY	hsa05332:Graft-versus-host disease	10	6.85	1.70E-07	10.96	1.48E-05	8.68E-07	1.83E-04
KEGG_PATHWAY	hsa05223:Non-small cell lung cancer	11	7.53	3.13E-07	8.70	2.72E-05	1.51E-06	3.38E-04
KEGG_PATHWAY	hsa04940:Type I diabetes mellitus	10	6.85	3.37E-07	10.17	2.93E-05	1.54E-06	3.64E-04
KEGG_PATHWAY	hsa04910:Insulin signaling pathway	16	10.96	3.71E-07	5.06	3.23E-05	1.61E-06	4.01E-04
KEGG_PATHWAY	hsa05212:Pancreatic cancer	12	8.22	6.03E-07	7.12	5.24E-05	2.50E-06	6.51E-04
KEGG_PATHWAY	hsa04510:Focal adhesion	19	13.01	6.21E-07	4.04	5.41E-05	2.46E-06	6.72E-04

KEGG_PATHWAY	hsa04930:Type II diabetes mellitus	10	6.85	9.33E-07	9.09	8.12E-05	3.53E-06	1.01E-03
KEGG_PATHWAY	hsa05200:Pathways in cancer	24	16.44	1.21E-06	3.13	1.05E-04	4.38E-06	1.30E-03
KEGG_PATHWAY	hsa05120:Epithelial cell signaling in Helicobacter pylori infection	11	7.53	2.89E-06	6.91	2.51E-04	1.01E-05	3.12E-03
KEGG_PATHWAY	hsa04640:Hematopoietic cell lineage	12	8.22	3.71E-06	5.96	3.23E-04	1.24E-05	4.01E-03
KEGG_PATHWAY	hsa05211:Renal cell carcinoma	11	7.53	3.79E-06	6.71	3.30E-04	1.22E-05	4.10E-03
KEGG_PATHWAY	hsa04210:Apoptosis	12	8.22	4.17E-06	5.89	3.62E-04	1.29E-05	4.50E-03
KEGG_PATHWAY	hsa05215:Prostate cancer	12	8.22	5.22E-06	5.76	4.54E-04	1.57E-05	5.65E-03
KEGG_PATHWAY	hsa05320:Autoimmune thyroid disease	9	6.16	1.83E-05	7.54	1.59E-03	5.32E-05	1.98E-02
KEGG_PATHWAY	hsa05222:Small cell lung cancer	11	7.53	2.01E-05	5.60	1.75E-03	5.63E-05	2.17E-02
KEGG_PATHWAY	hsa05213:Endometrial cancer	9	6.16	2.13E-05	7.40	1.85E-03	5.78E-05	2.30E-02
KEGG_PATHWAY	hsa04960:Aldosterone-regulated sodium reabsorption	8	5.48	3.54E-05	8.34	3.08E-03	9.34E-05	3.83E-02
KEGG_PATHWAY	hsa05210:Colorectal cancer	10	6.85	1.23E-04	5.09	1.06E-02	3.14E-04	1.33E-01
KEGG_PATHWAY	hsa05340:Primary immunodeficiency	7	4.79	1.29E-04	8.55	1.11E-02	3.20E-04	1.39E-01
KEGG_PATHWAY	hsa04920:Adipocytokine signaling pathway	9	6.16	1.38E-04	5.74	1.19E-02	3.33E-04	1.49E-01
KEGG_PATHWAY	hsa04810:Regulation of actin cytoskeleton	15	10.27	4.01E-04	2.98	3.43E-02	9.42E-04	4.32E-01
KEGG_PATHWAY	hsa04672:Intestinal immune network for IgA production	7	4.79	8.51E-04	6.10	7.14E-02	1.95E-03	9.16E-01
KEGG_PATHWAY	hsa04514:Cell adhesion molecules (CAMs)	11	7.53	8.90E-04	3.56	7.45E-02	1.98E-03	9.57E-01
KEGG_PATHWAY	hsa04070:Phosphatidylinositol signaling system	8	5.48	1.51E-03	4.62	1.23E-01	3.28E-03	1.62E+00
KEGG_PATHWAY	hsa04060:Cytokine-cytokine receptor interaction	15	10.27	2.75E-03	2.45	2.13E-01	5.82E-03	2.93E+00
KEGG_PATHWAY	hsa04914:Progesterone-mediated oocyte maturation	8	5.48	3.58E-03	3.97	2.68E-01	7.41E-03	3.81E+00
KEGG_PATHWAY	hsa05218:Melanoma	7	4.79	5.76E-03	4.21	3.95E-01	1.16E-02	6.06E+00
KEGG_PATHWAY	hsa04150:mTOR signaling pathway	6	4.11	6.73E-03	4.93	4.44E-01	1.33E-02	7.04E+00

KEGG_PATHWAY	hsa04912:GnRH signaling pathway	8	5.48	7.35E-03	3.49	4.73E-01	1.42E-02	7.66E+00
KEGG_PATHWAY	hsa04621:NOD-like receptor signaling pathway	6	4.11	1.40E-02	4.14	7.06E-01	2.62E-02	1.41E+01
KEGG_PATHWAY	hsa05416:Viral myocarditis	6	4.11	2.38E-02	3.61	8.77E-01	4.37E-02	2.30E+01
KEGG_PATHWAY	hsa04622:RIG-I-like receptor signaling pathway	6	4.11	2.38E-02	3.61	8.77E-01	4.37E-02	2.30E+01
KEGG_PATHWAY	hsa05322:Systemic lupus erythematosus	7	4.79	2.68E-02	3.02	9.06E-01	4.80E-02	2.54E+01
KEGG_PATHWAY	hsa04360:Axon guidance	8	5.48	2.96E-02	2.65	9.27E-01	5.19E-02	2.77E+01
KEGG_PATHWAY	hsa04010:MAPK signaling pathway	11	7.53	8.99E-02	1.76	1.00E+00	1.51E-01	6.39E+01
